# Biochemical and molecular characterization of the isocitrate dehydrogenase with dual coenzyme specificity from the obligate methylotroph *Methylobacillus Flagellatus*

**DOI:** 10.1371/journal.pone.0176056

**Published:** 2017-04-19

**Authors:** Anastasia Y. Romkina, Michael Y. Kiriukhin

**Affiliations:** Ajinomoto-Genetika Research Institute, Moscow, Russia; Russian Academy of Medical Sciences, RUSSIAN FEDERATION

## Abstract

The isocitrate dehydrogenase (*Mf*IDH) with unique double coenzyme specificity from *Methylobacillus flagellatus* was purified and characterized, and its gene was cloned and overexpressed in *E*. *coli* as a fused protein. This enzyme is homodimeric,—with a subunit molecular mass of 45 kDa and a specific activity of 182 U mg ^-1^ with NAD^+^ and 63 U mg ^-1^ with NADP^+^. The *Mf*IDH activity was dependent on divalent cations and Mn^2+^ enhanced the activity the most effectively. *Mf*IDH exhibited a cofactor-dependent pH-activity profile. The optimum pH values were 8.5 (NAD^+)^ and 6.0 (NADP^+^).The K_m_ values for NAD^+^ and NADP^+^ were 113 μM and 184 μM respectively, while the K_m_ values for DL-isocitrate were 9.0 μM (NAD^+^), 8.0 μM (NADP^+^). The *Mf*IDH specificity (k_cat_/K_m_) was only 5-times higher for NAD^+^ than for NADP^+^. The purified *Mf*IDH displayed maximal activity at 60°C. Heat-inactivation studies showed that the *Mf*IDH was remarkably thermostable, retaining full activity at 50°C and losting ca. 50% of its activity after one hour of incubation at 75°C. The enzyme was insensitive to the presence of intermediate metabolites, with the exception of 2 mM ATP, which caused 50% inhibition of NADP^+^-linked activity. The indispensability of the N^6^ amino group of NAD(P)^+^ in its binding to *Mf*IDH was demonstrated. *Mf*IDH showed high sequence similarity with bacterial NAD(P)^+^-dependent type I isocitrate dehydrogenases (IDHs) rather than with eukaryotic NAD^+^-dependent IDHs. The unique double coenzyme specificity of *Mf*IDH potentially resulted from the Lys340, Ile341 and Ala347 residues in the coenzyme-binding site of the enzyme. The discovery of a type I IDH with double coenzyme specificity elucidates the evolution of this subfamily IDHs and may provide fundamental information for engineering enzymes with desired properties.

## Introduction

Isocitrate dehydrogenase (IDH) is a key enzyme in the tricarboxylic acid (TCA) cycle that catalyzes the oxidative decarboxylation of isocitrate, which is accompanied by the reduction of NAD(P)^+^ to NAD(P)H, to yield α-ketoglutarate that is used for biosynthesis. This enzyme belongs to the large and ancient β-decarboxylating dehydrogenase superfamily and plays central roles in energy metabolism, glutamate/amino acid biosynthesis and vitamin production[1,2]. Due to their central role in metabolism, IDHs are distributed throughout Archaea, Bacteria, and Eukarya [3]. Two types of IDHs are distributed based on their coenzymes: NAD^+^-specific IDH (EC 1.1.1.41, NAD-IDH) and NADP^+^-specific IDH (EC 1.1.1.42, NADP-IDH). Three types of IDHs can be distinguished by other criteria: type I IDHs (NAD^+^ and NADP^+^), type II homodimeric IDHs (NADP^+^-specific) and monomeric IDHs (NADP^+^-specific). Recently, novel type II homodimeric NAD-IDHs from *O*. *lucimarinus*, *Micromonas sp*. and *C*. *litoralis*,- and novel monomeric NAD-IDHs from *Campylobacter sp*. were discovered [3,4]. Prokaryotes usually have one IDH, whose dependence on NADP^+^ or NAD^+^ is correlated with the presence or absence of a glyoxylate bypass in the organism; however, some organisms, such as *M*. *tuberculosis*, *P*. *psychrophila* or *Vibrio sp*., have two structurally different isozymes [1,5–7]. For example, both *C*. *psychrerythraea* and *X*. *campestris* have one homodimeric type I IDH and one monomeric IDH with different biochemical properties [8–10]. Most prokaryotic IDHs that have been investigated are NADP^+^-dependent and homodimeric [11–17]. A few NADP^+^-dependent monomeric IDHs [9,18–20] and homotetrameric IDH from *T*. *maritima* [17] have also been characterized. Recently, numerous prokaryotic and archaeal homodimeric NAD^+^-IDHs and, a few monomeric NAD^+^-IDHs, have been reported [21–27]. However, NAD^+^-dependency is relatively rare in prokaryotic IDHs, and true double coenzyme specificity has never been reported. One common feature shared by these prokaryotes is that they lack a complete TCA cycle due to the absence of an α-ketoglutarate dehydrogenase [24]. The exact functions of prokaryotic NAD^+^-IDHs are still unclear.

Insufficient data exist regarding IDHs from methylotrophic bacteria. Lloyd and Weitzman, demonstrated that an IDH from the obligate methylotroph *M*. *methylotrophusis* is NAD^+^-linked [28]. Two isozymes, one that is NAD^+^-dependent and one that is NADP^+^-dependent, were partially purified from the obligate methylotroph *Pseudomonas* W6 [29]. The facultative methylotroph *P*. *oleovorans* possessed only an NAD^+^-specific IDH [30].

*Methylobacillus flagellatus* is an obligate methylotroph with the 2-keto-3-deoxy-6-phosphogluconate aldolase/transaldolase variant of the ribulose monophosphate (RuMP) pathway of formaldehyde fixation [31]. This organism has an incomplete TCA cycle, and lacks - α-ketoglutarate dehydrogenase [32]. Thus, the IDH of *M*. *flagellatus* provides α-ketoglutarate, which participates in NH_4_^+^ fixation following the reaction catalysed by NADP-dependent glutamate dehydrogenase. In *M*. *flagellatum* this enzyme as well as citrate synthase are not regulated by intermediary metabolites [33–35]. NH_4_^+^ assimilation might be regulated only at the level of the reactions in the RuMP cycle or by the modulation of IDH activity [35]. Although only one gene encoding an IDH was found in the genome of *M*. *flagellatus*, we demonstrated that NAD^+^-IDH activity is up-regulated and NADP^+^-IDH activity is down-regulated in the N-limited cultures [33].

In this paper, we report the purification, cloning, heterologous expression, and characterization of the IDH from *M*. *flagellatus* (*Mf*IDH). We provide experimental evidence that demonstrates that *Mf*IDH is an enzyme with bona fide double cofactor specificity and its catalytic efficiency with NAD^+^ (k_cat_/K_m_) is comparable to the efficiency of prokaryotic NADP^+^-IDHs. This detailed enzymatic characterization of *Mf*IDH adds a new and interesting member to the IDH family.

## Materials and methods

### Bacterial strains and growth conditions

*Methylobacillus flagellatus* ATCC51484 was obtained from the laboratory collection and was aerobically grown at 42°C in minimal medium, containing 2% (v/v) methanol [31]. The genomic DNA was isolated with a Promega Wizard kit.

### Preparation of cell-free extracts and purification of the native enzyme

All procedures were carried out at 4°C. Cells were harvested by centrifugation, washed with an equal volume of 50 mM potassium phosphate buffer (PPB), pH 7.5, and centrifuged again. The pellet was resuspended in an appropriate amount of the same buffer and disrupted by sonication. The cell debris was removed by centrifuging the sample for 30 min at 10 000×g. The supernatant was heat treated for 30 min at 55°C and rapidly cooled. The precipitated protein was removed by centrifugation. Solid (NH_4_)_2_SO_4_ was added to the heat-treated extract until the solution reached 50% saturation. The protein that precipitated was removed by centrifugation and discarded. The solution concentration of (NH_4_)_2_SO_4_ was increased to 80% saturation, and the resulting protein precipitate was collected by centrifugation and redissolved in a minimal volume of 50 mM Tris-HCl buffer, pH 7.0 (buffer A). This enzyme solution was loaded onto an S-200 Sephacryl column (90×2.6 cm), that was equilibrated with buffer A. Active fractions, wich were eluted with buffer A, were pooled and loaded onto a Red Sepharose CL-6B (Pharmacia Biotech) column (15×1.6 cm), that had been previously equilibrated with buffer A. The enzyme was eluted with a linear KCl gradient (0–1 M during 15 column volumes). The active fractions were pooled and concentrated, and their buffer was changed to a 25 mM histidine-HCl buffer, pH 6.4 (buffer B) by using a Vivaspin 20 centrifugal concentrator. The enzyme solution was loaded onto a Polybuffer exchanger PBE94 (Pharmacia Biotech) column (30×1.0 cm), that had been previously equilibrated with buffer B. The column was washed with buffer B (one column volume) and then it contents were eluted with 11 column volumes of Polybuffer 74, pH 5.0. The active fractions were pooled and concentrated, and their buffer was changed to 25 mM Tris-HCl, pH 7.2, by using a centrifugal concentrator. The results of a typical purification procedure are reported in S1 Table.

### Plasmid construction

Based on the genome sequence of *M*. *flagellatus* **ATCC**51484 (GenBank accession no. CP000284) [32], two specific primers were designed to amplify the complete IDH gene: sense primer

5′-GCGCGCCATGGGCAGCAGCCATCATCATCATCATCACAGCAGCGGCATGTCTACAAAAATCAAAGTACCCACTACTG-3′ (NcoI site underlined) and antisense primer

5′-CCATTGGATCCTGACATGTGCTTGACGATCTCCGCACCGAATTCTGCACTGC-3′

(BamHI site underlined). The expression vector pET-15b was used for the heterologous expression of *Mf*IDH. The PCR product containing the IDH gene was purified, digested and ligated into the NcoI/BamHI-digested multiple cloning site of pET-15b; in this manner, the plasmid region encoding the thrombin recognition site was eliminated, creating the recombinant plasmid pET-*Mf*IDH. The presence of the *Mf*IDH gene with a 6x His-tag coding sequence directly downstream of the start codon was confirmed by sequencing.

### Overexpression and purification

The *E*. *coli* BL21 (DE3) strain harboring the pET-*Mf*IDH plasmid was cultured overnight in Luria–Bertani (LB) medium supplemented with 150 μg/mL of ampicillin at 37°C. The cells were then inoculated into 100 mL of fresh LB (with the same antibiotic) to a final OD_600nm_ of up of 0.1 and grown until the cell density reached an OD_600nm_ of 0.5–0.6. At this time, IPTG was added to the culture at a final concentration of 1 mM; the incubation continued for 3 more hours. The cells were harvested and resuspended in sonication buffer. The cell debris was then removed by centrifuging the sample at 12 000× g for 15 min at 4°C. The recombinant *Mf*IDH with the 6x-His -tag on its N-terminus was purified using Ni-NTA Affinity Resin (Clontech, La Jolla, CA) according to the manufacturer’s instructions. The protein fractions were eluted with an imidazole gradient from 30 mM (in the binding buffer) to 500 mM (in the elution buffer). The fraction containing the recombinant *Mf*IDH was dissolved in a buffer with 50 mM Tris-HCl (pH 7.5) and 10% glycerol. The purity of the recombinant enzyme was analyzed and confirmed using 12% SDS-PAGE. For the Western blot analysis, the SDS-PAGE gels were transferred to a nitrocellulose membrane by electroblotting. The membrane was blocked for 1 h at room temperature with 5% skim milk in a buffer containing 50 mM Tris-HCl (pH 7.5), 150 mM NaCl, and 0.2% Tween-20. His-tagged polyclonal antibody (Thermo Fisher Scientific, USA) and alkaline phosphatase-conjugated goat anti-rabbit IgG (Promega, USA) were applied to the blot, at the appropriate dilution. The chemiluminescence signal was visualized by exposing the blots to X-ray film.

### Measurement of enzyme activity

The IDH activity was routinely measured by monitoring the reduction of NAD^+^ (or NADP^+^) at 340 nm. The reaction mixtures were incubated at 42°C and contained 100 mM Tris-HCl buffer (pH 7.5), 2 mM MnCl_2_, 5.0 mM DL-isocitrate, 0.4 mM NAD^+^ or NADP^+^ and the enzyme, with a total volume of 1.0 mL. After determing their pH optima, NAD^+^-IDH activity was measured in 100 mM Tris-HCl buffer (pH 8.5), and NADP^+^-IDH activity was measured in 100 mM Bis-Tris-HCl buffer (pH 6.0). The increase in NAD(P)H concentration was determined by monitoring the absorbance at 340 nm with a thermostated Shimadzu-1800 UV-Vis spectrophotometer (Shimadzu Corp, Japan) and converting the absorbance to concentration using a molar extinction coefficient of 6.22 mM^-1^ cm^-1^. One unit (U) of activity was defined as 1 μmol of NAD(P)H formed per min. The protein concentrations were determined using the Bio-Rad protein assay kit (Bio-Rad, USA) with bovine serum albumin as the standard. All the reported values are the means of at least three independent experiments.

### Characterization of the native and recombinant *Mf*IDHs

The molecular mass of the native and recombinant *Mf*IDH was estimated using gel filtration chromatography with a HiLoad 10/300 Superdex 200 column (GE Healthcare), equilibrated with 0.05 M PPB (pH 7.0) containing 0.15 M NaCl and 0.01% sodium azide. The protein standards used to calibrate the molecular weights determined from the gel were carbonic anhydrase (29 kDa), albumin (66 kDa), alcohol dehydrogenase (150 kDa), β-amylase (200 kDa), apoferritin (443 kDa) and thyroglobulin (669 kDa).

The effects of pH and temperature on the native *Mf*IDH activity were determined in the presence of Mn^2+^. To obtain its pH profile, the enzyme’s activity was assayed in 100 mM buffer (Bis-Tris-HCl, pH 5.0–7.0, Tris–HCl, pH 7.5–9.0 or 2-amino-2-methyl-1,3-propanediol, pH 9.5–10.5). The effect of the temperature on the activity was determined for temperatures of up to 65°C. The influence of temperature on protein stability was investigated by incubating the pure enzyme (0.07 mg/ml) in 50 mM PPB at different temperatures for 60 min. Next, the aliquots were immediately cooled on ice and then their residual activity was assayed. The kinetic parameters for the native *Mf*IDH were determined by measuring the activity of native *Mf*IDH at various concentrations of one substrate (isocitrate and NADP^+^ or NAD^+^) while at saturating concentrations of the other substrate. The apparent kinetic parameters were derived from a double-reciprocal Lineweaver-Burk plot.

The effects of different metal ions (2 mM MnCl_2_, 2 mM MgCl_2_, 2 mM CaCl_2_, 2 mM CuSO_4_, and 2 mM ZnSO_4_), metabolites or cofactor analogs on the native/recombinant activity were determined using the standard assay protocol.

### Polyacrylamide gel-electrophoresis

12% SDS-PAGE and non-denaturing gradient polyacrylamide (4–20%, w/v) electrophoresis were carried out as described elsewhere. The IDH activity after electrophoresis was measured by incubating the gel slices in a solution that, stains based on the enzyme activity: 100 mM Tris-HCl buffer (pH 7.5), 2 mM MnCl_2_, 5.0 mM DL-isocitrate, 2.0 mM NAD^+^ or NADP^+^, 1.0 mM Nitroblue tetrazolium, and 0.5 mM phenazine methosulfate in 100 mM Tris-HCl buffer, pH 7.5. Isoelectric focusing experiments were performed in a horizontal slab gel. Pharmalyte 3–10 (GE Healthcare) was used to obtain a pH gradient.

### Structure-based protein sequence alignment

X-ray crystal structures of *A*. *thiooxidans* NAD-IDH (*At*IDH, 2D4V), *E*. *coli* NADP-IDH (*Ec*IDH, 9ICD) and B. *subtilis* NADP-IDH (*Bs*IDH, 1HQS) were downloaded from the PDB database (http://www.rcsb.org/pdb/home/home.do). The homology models of *Z*. *mobilis* NAD-IDH (*Zm*IDH) and *M*.*flagellatus* IDH (*Mf*IDH) were generated using the SWISS-MODEL modeling server (http://swissmodel.expasy.org). The structure based amino acid sequence alignment was made using the CLUSTALX program (ftp://ftp.ebi.ac.uk/pub/software/clustalw2) and the ESPRIPT 3.0 web tool (http://espript.ibcp.fr/ESPript/ESPript/) [36,37].

## Results and discussion

### Enzyme purification and characteristics

**Fig 1:**
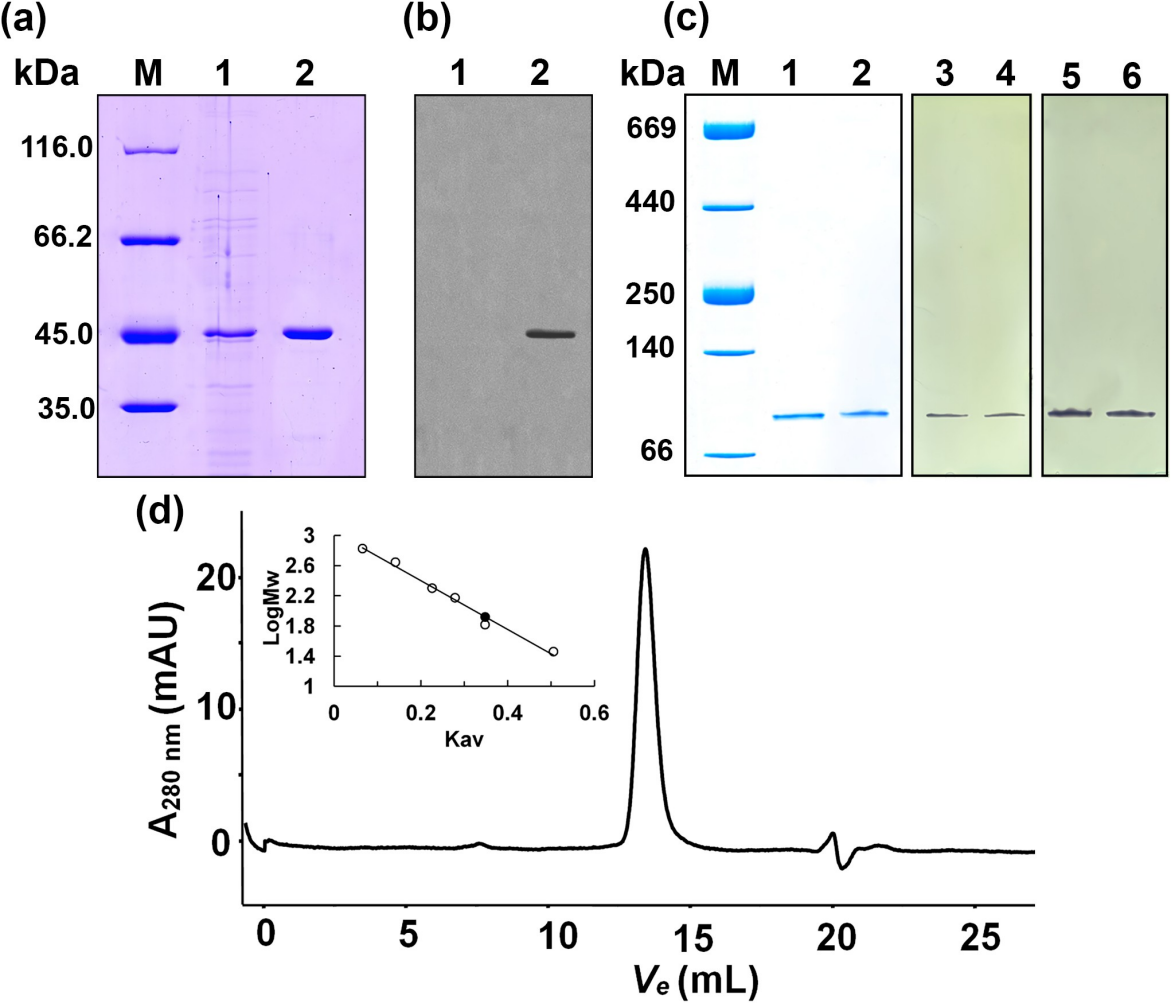
Overexpression, purification and oligomeric state determination of the recombinant *Mf*IDH. (a) The protein purity was determined using 12% SDS-PAGE. M, protein marker; lane 1, crude extracts of cells harboring plasmid pET-*Mf*IDH after induction with IPTG; lane 2, purified protein. (b) Detection of *Mf*IDH by Western blot using the anti-6×His antibody as a probe. Lane 1, negative control, crude extracts of cells harboring pET-15b(+) with IPTG induction; lane 2, purified protein. (c) Gradient non-denaturing PAGE. M, protein marker; lane 1, purified native *Mf*IDH; lane 2, purified recombinant *Mf*IDH; Zymogram assay of the purified proteins. Staining for the NADP^+^-dependent activity: lane 3, native *Mf*IDH, lane 4, recombinant *Mf*IDH. Staining for the NAD^+^-dependent activity: lane 5, native *Mf*IDH, lane 6, recombinant *Mf*IDH. (d) Molecular mass determination using gel filtration chromatography. The flow rate was 0.5 mL min^-1^, and the proteins were detected by monitoring their absorbance at 280 nm. The molecular mass standard curve is inset. The measurement of the recombinant *Mf*IDH is represented as a dark dot (●). The standard proteins are represented as open circles (○) and are carbonic anhydrase (29 kDa), albumin (66 kDa), alcohol dehydrogenase (150 kDa), β-amylase (200 kDa), apoferritin (443 kDa) and thyroglobulin (669 kDa). The *V*_*e*_ of the recombinant *Mf*IDH is 13.36 mL.

The native and the recombinant 6x-His-tagged *Mf*IDHs have essentially the same biochemical characteristics; the native enzyme was used in this study unless otherwise noted. A 232-fold purification of native *Mf*IDH was achieved with a 46% recovery. Polyacrylamide gel electrophoresis in denaturing and non-denaturing conditions showed that the native and recombinant *Mf*IDH were purified to homogeneity. A single protein band with a molecular mass of approximately 45.0 kDa was observed on SDS-PAGE gels, was correlated well with the predicted value (44.6 kDa) (Fig 1A), which was also recognized as His-tag protein in the Western blot (Fig 1B). After staining them for enzymatic activity, the gradient non-denaturing PAGE gels exhibited one protein band with a molecular mass of approximately 87 kDa, suggesting a homodimeric structure of the native enzyme (Fig 1C). Size exclusion chromatography (SEC) also confirmed that *Mf*IDH is a homodimer in solution. A single symmetric peak with a molecular mass of approximately 83.6 kDa was observed (Fig 1D) in the gel filtration experiment, while the calculated molecular mass of homodimeric *Mf*IDH was 89.2 kDa.

An isoelectric focusing gel exhibited one protein band with a pI of 5.5, which was lower than the predicted (pI 5.9) (S1 Fig).

The specific activity of the purified *Mf*IDH was 182 U mg ^-1^ when using NAD^+^ and 63 U mg ^-1^ when using NADP^+^ as substrates,demonstrating the enzyme possesses true double coenzyme specificity. The enzyme exhibits Michaelis–Menten kinetics. The enzyme’s apparent K_m_ value for DL-isocitrate was 9.0 μM when NAD^+^ was used as a cosubstrate and 8.0 μM when NADP^+^ was used. The kinetic analysis showed that the K_m_ values of *Mf*IDH for NAD^+^ and NADP^+^ were almost equal—113 μM and 184 μM, respectively. The *Mf*IDH specificities [(k_cat_/K_m_)NAD/(k_cat_/K_m_)NADP] were only 5-times higher for NAD^+^ than for NADP^+^ (Table 1). Based on this observation, *Mf*IDH showed slight preference for NAD^+^, indicating that *Mf*IDH was wrongly annotated as an NADP^+^-dependent enzyme in GenBank. However, our results provide solid experimental evidence that this enzyme demonstrates almost equal cofactor specificity, with a slight preference toward NAD^+^.

**Table 1:** Kinetic parameters on the activity of *Mf*IDH.

NAD^+^			NADP^+^			
*K*_*m*_ (μM)	*k*_*cat*_ (s^-1^)	*k*_*cat*_*/K*_*m*_ (μM^-1^s^-1^)	*K*_*m*_ (μM)	*k*_*cat*_ (s^-1^)	*k*_*cat*_*/K*_*m*_ (μM^-1^s^-1^)	Specificity (*k*_*cat*_*/K*_*m*_) ^NAD^/(*k*_*cat*_*/K*_*m*_) ^NADP^
113	166	1.5	184	56	0.3	5

According to Zhu et al, NAD^+^ usage is an ancestral trait and NADP^+^ dependency by prokaryotic IDHs emerged near the time that eukaryotic mitochondria first appeared, (some 3.5 billion years ago). The switch of the coenzyme specificity of prokaryotic IDH from NAD^+^ to NADP^+^ is an ancient adaptation to the anabolic demand for NADPH during growth on acetate [1]. The aerobic Gram-negative bacterium *M*. *flagellatus* which has an uncoupled TCA cycle contains an IDH that is specific for both NAD^+^ and NADP^+^, which provides flexibility to use either available cofactor and generate NADH or NADPH.

The K_m_ value of *Mf*IDH for NAD^+^ (113 μM) is higher than that determined for *P*. *furiosus* NAD^+^-IDH (68 μM) [23], but lower than those of *Z*. *mobilis* NAD^+^-IDH (245 μM), *S*. *suis* NAD^+^-IDH (233 μM), *A*. *thiooxidans* NAD^+^-IDH (184 μM), *S*.*mutans* NAD^+^-IDH (154 μM), and *M*. *capsulatus* NAD^+^-IDH (122 μM) [16].

The K_m_ value of *Mf*IDH for NADP^+^ (184 μM) is much higher than those of most homodimeric or monomeric IDHs, such as *B*. *subtilis* NADP^+^-IDH (15 μM) [38], *E*. *coli* NADP^+^-IDH (17 μM) [39], *P*. *nautica* NADP^+^-IDH (25 μM), and *S*. *diastaticus* NADP^+^-IDH (8.5 μM) [18], but is in the range of those of *H*. *volcanii* NADP^+^-IDH (101 μM) [40] and *H*. *pylori* NADP^+^-IDH (176 μM) [15]. The K_m_ value of *Mf*IDH for DL-isocitrate (8–9 μM) is within the range observed for many characterized IDHs [18].

Although the NAD^+^-linked *Mf*IDH activity has a lower cofactor affinity than its NADP^+^- dependent counterparts, its catalytic efficiency (1.5 μM^-1^ s^-1^) is very close to those of the IDHs from *E*. *coli* IDH (4.7 μM^-1^s^-1^) and *B*. *longum* (1.87 μM^-1^s^-1^), and is higher than those of the NAD^+^-linked IDHs from *Z*. *mobilis* IDH (0.46 μM^-1^s^-1^) and *A*. *thiooxidans* IDH (0.25 μM^-1^s^-1^) [11,26]. In contrast, the catalytic efficiency of the NADP^+^-linked activity of *Mf*IDH (0.3 μM^-1^s^-1^) is much lower and comparable with the efficiency of NAD^+^-dependent homodimeric IDHs.

### Sequence analysis

**Fig 2:**
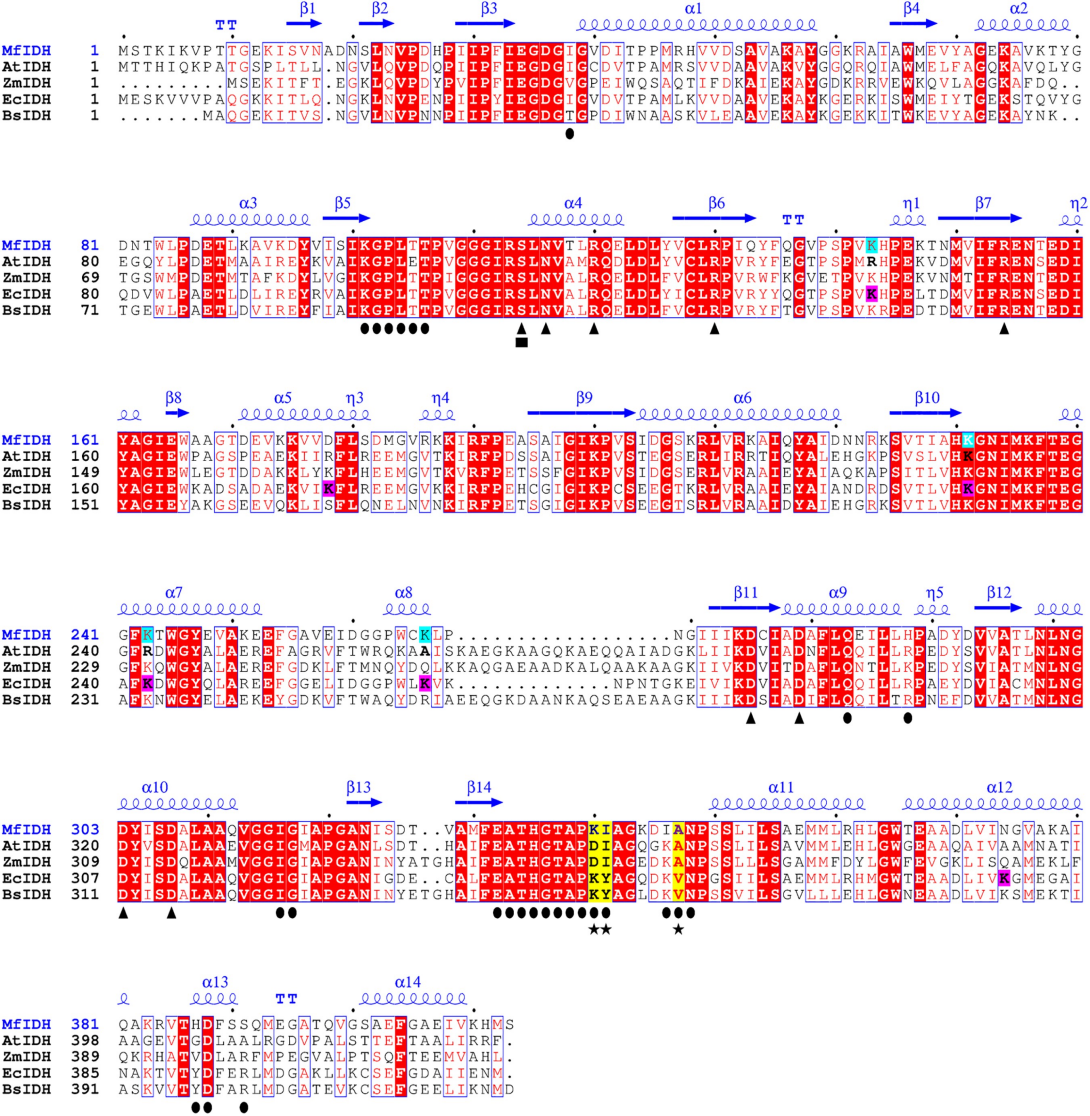
Structure-based sequence alignment of *Mf*IDH with other dimeric IDHs. High-resolution crystal structures of the *A*. *thiooxidans* NAD-IDH (*At*IDH, 2D4V), *B*. *subtilis* NADP-IDH (*Bs*IDH, 1HQS) and *E*. *coli* NADP-IDH (*Ec*IDH, 9ICD) were downloaded from the PDB database. The *Mf*IDH model of the IDH from *M*. *flagellatus* and the *Zm*IDH model of the NAD-IDH from *Z*. *mobilis* were generated using the SWISS-MODEL modeling server with *At*IDH structure as the template. The secondary structure of *Mf*IDH is depicted above the alignment. The completely conserved amino acids are highlighted as shaded red boxes. The conserved residues involved in cofactor- (●) and substrate-binding (▲) are indicated, respectively. The conserved phosphorylation sites are indicated (■). The lysine residues that may be acetylated in *Mf*IDH and *Ec*IDH are highlighted with shades light-blue and pink boxes, respectively. The major cofactor specificity determinants are highlighted with shaded yellow boxes and indicated with stars (★). The alignment was drawn with ESPRIPT 3.0.

The IDH gene in *M*.*flagellatus* (*Mf*IDH) is 1242 bp in length and encodes a polypeptide of 413 amino acids. The overall GC content is approximately 56.17% (genome 55.7%), which is similar to those of the chromosomes of *Methylophilaceae* species (37–57%) [41]. The search for regions that are identical to the *Mf*IDH gene indicated that the highest identity values were with IDHs from the following organisms: *Methylobacillus glycogenes* (96%), *Methylovorus glucosotrophus* (91%), *Methylotenera mobilis* (87%), *Candidatus Methylopumilus turicensis* (86%), *Methylotenera versatilis* (86%), and *Methylophilus methylotrophus* (86%). The amino acid identities of *Mf*IDH with typical homodimeric NADP^+^-IDHs from *E*. *coli* and *B*. *subtilis*, and with NAD^+^-IDHs from *A*. *thiooxidans* and *Z*. *mobilis* were 66, 62, 58 and 56%, respectively. The 3D-structure of *Mf*IDH was modeled using the *At*IDH (2D4V) structure as a template. A secondary- structure-based alignment revealed that most structural elements that are, involved in the binding of the substrate and coenzyme are highly conserved within prokaryotic homodimeric type I IDHs (Fig 2).

The interactions between the 2’-phosphate of NADP^+^ and the amino acid residues Lys344, Tyr345 and Val351 in *Ec*IDH, and Lys350, Tyr351 and Val357 in *Bs*IDH have been declared the determinants of NADP^+^ cofactor specificity [1,42]. The possibility of switching the cofactor preference was shown experimentally by replacement of the original motif Lys350, Tyr351 and Val357 in NADP^+^-IDH of *E*.*coli* with the mutated motif Asp350, Ile351 and Ala357 in engineered NAD^+^-IDH of *E*.*coli* [42]. According to Dean and Golding, Imada et al; substitution of the Lys with an Asp, results in the formation of double hydrogen bonds with the 2′- and 3′-hydroxyl groups of the adenosine ribose of NAD^+^ and the repelling of the negatively charged 2’-phosphate of NADP^+^ through electrostatic repulsion, which together cause the NAD^+^ cofactor specificity of *At*IDH (Asp357, Ile358 and Ala364) and *Zm*IDH (Asp348, Ile349 and Ala355) [42,43].

Furthermore, the amino acid residues Asp328, Ile329 and Ala335 have been declared the determinants of NAD cofactor specificity in NAD-IDH from *Pyrococcus furiosus* [23]. The *s*ite-directed mutagenesis experiment that replaced Asp328 with Lys328 in the cofactor discrimination site of the NAD^+^-IDH from *P*. *furiosus;* led to a significant reduction in K_m_ for NADP (~27fold), whereas the K_m_ for NAD was unaltered and the specificity for NADP was increased five-fold compared with the wild-type enzyme. This motif–Lys328, Ile329 and Ala335 results in a double coenzyme specificity of chimeric *P*.*furiosus* IDH. The introduction of the double replacement of Asp-328–Lys/Ile-329–Tyr (motif Lys328, Tyr329 and Ala347) has not changed the efficiency of NADP-IDH, but rather slightly increased both K_m_ and K_cat_ for NADP. The k_cat_ was unaltered compared with the single-mutated enzyme [23]. The structure-based alignment revealed that there is the same motif—Lys340, Ile341 and Ala347 in naturally occurred *Mf*IDH. Thus, signature residues involved in substrate discrimination in *Mf*IDH appeared to be Lys340, Ile341 and Ala347 (Fig 2). The ability to use efficiently both NAD^+^ and NADP^+^ as cofactors is caused by the presence of these three key amino acids in the protein structure of *Mf*IDH. The *Mf*IDH was incorrectly annotated as NADP^+^ specific isocitrate dehydrogenase. We suggest that *Mf*IDH can be annotated as a homodimeric type I isocitrate dehydrogenase with dual coenzyme specificity.

Post-translational modifications are one of the most efficient biological mechanisms for regulating enzyme activity and cellular physiology. The activity of *Ec*IDH is regulated by an IDH-kinase/phosphatase (*aceK*) that responds to changes in the metabolic environment [44]. Although phosphorylation sites are conserved in *Ec*IDH (Ser113), *At*IDH (Ser113), *Bs*IDH (Ser104), *Zm*IDH (Ser102) and *Mf*IDH (Ser114), no corresponding IDH-kinase/phosphatase gene was found in the genome of *M*. *flagellatus*.

It was recently found that lysine acetylation [45–47] as well as succinylation [48,49] activities are abundant in *E*. *coli* and might be involved in modifying or regulating the activities of enzymes involved in the synthesis of building blocks in response to environmental changes and critical metabolic processes. Six acetylation sites were found in *Ec*IDH [47]. Zhang et al, by mimic mutagenesis demonstrated that both Lys100 and Lys242 are important for the activity of *Ec*IDH and that lysine succinylation is likely to inhibit or abolish its enzymatic function [48]. Analog sites corresponding to the lysine acetylation sites of *Ec*IDH (Lys142, Lys177, Lys230, Lys242, Lys265, Lys378) [47] are also found in *Mf*IDH (Lys143, Lys231, Lys243, Lys266). Analogues lysine succinylation sites of *Ec*IDH (Lys100, Lys186, Lys199, Lys230, Lys235, Lys242, Lys387) [49] were also conserved in *Mf*IDH (Lys101, Lys187, Lys200, Lys231, Lys236, Lys243, Lys383) (Fig 2), although there is no evidence that *Mf*IDH can be regulated by acetylation or succinylation in vivo.

### Effects of pH and temperature

**Fig 3:**
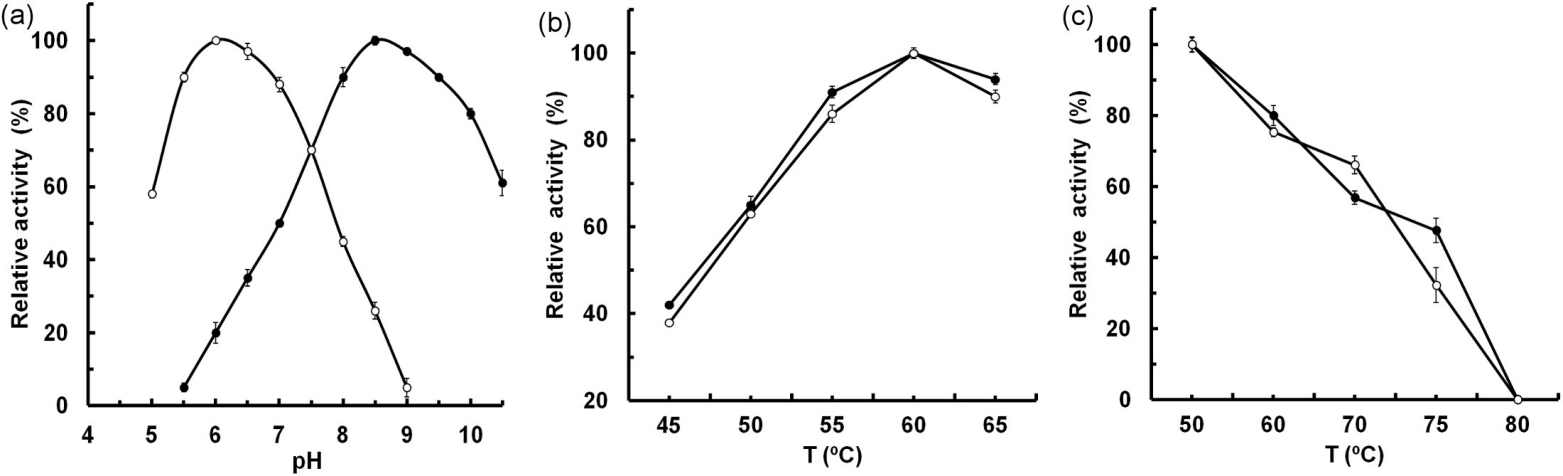
Effects of pH and temperature on the activity of *Mf*IDH. (a) The effects of pH on the NAD^+^-dependent (●) and NADP^+^-dependent (○) activities of *Mf*IDH from pH 5.0 to 10.0 in the presence of Mn^2+^. (b) The effects of temperature on NAD^+^-dependent (●) and NADP^+^-dependent (○) activities of *Mf*IDH from 45 to 65°C. (c) Heat-inactivation profiles of NAD^+^-dependent (●) and NADP^+^-dependent (○) activities of *Mf*IDH incubated at 50 to 80°C. The incubation time is 60 min.

The effects of the pH on the *Mf*IDH activity were determined for the NAD^+^- and NADP^+^-linked reactions in the presence of Mn^2+^. Surprisingly, *Mf*IDH exhibited a strict cofactor-dependent pH-activity profile, which has never been described in the literatur. The results demonstrate that the optimum pH is 8.5 with NAD^+^ and 6.0 with NADP^+^ (Fig 3A). For NAD^+^-linked activity, this value is similar to those of the *Z*. *mobilis* (pH 8.5) [26] and *A*. *thiooxidans* NAD^+^-IDHs (pH 8.5) [21], but is lower than that of the *H*. *thermophilus* NAD^+^-IDH (pH 10.5) [24]. For the NADP^+^-linked activity, this pH value is rather similar to that of the IDH from the acidophilic fungus *A*. *niger* (pH 6.0–8.0) [50]. The temperature for maximum activity *Mf*IDH is approximately 60°C, which is similar to those of the *B*. *longum* IDH (60°C) [51] and *L*. *interrogan* IDH (60°C) [12], but higher than that of the *E*. *coli* IDH (50°C) [22] (Fig 3B). Heat-inactivation studies revealed that the *Mf*IDH is remarkably thermostable, retaining its full activity at 50°C and losing ca. 50% of its activity after one hour of incubation at 75°C (Fig 3C). Thus, the thermostability of *Mf*IDH is closer to that of IDHs from thermophiles rather than mesophiles [52]. The increased thermostability of *Mf*IDH may be explained by its possessing twofold fewer Cys residues than *Ec*IDH does (0.70 and 1.40%, respectively); having fewer Cys residues is a common trend for thermophilic proteins [22]. The aromatic cluster in the clasp domain has previously been observed in the IDHs of hyperthermophilic *A*. *fulgidus* and *A*. *pernix*, and is believed to stabilize the interface [22]. The aromatic cluster of the *A*. *fulgidus* IDH contains Phe179, which is substituted by the nonpolar residue Met in typical mesophilic IDHs, e.g., Met183 (*AtI*DH), Met183 (*Ec*IDH) and Met172 (*Zm*IDH). Interestingly, *Mf*IDH has a polar residue (Gly184) at the same position (Fig 2), which is typical for methylotrophic IDHs, e.g., *M*. *glycogenes*, *M*. *glucosotrophus*, *M*. *mobilis*, *M*. *versatilis* and others. The role of Gly in the clasp stabilization at elevated temperature requires further investigation.

### Effects of metal ions on *Mf*IDH activity

The effects of different cations on the *Mf*IDH activity were studied, and the results indicate that *Mf*IDH retaines ca. 18% of its activity even without the addition of divalent ions (Table 2). This behavior is unusual for most IDHs, whose actvities entirely depend on the binding of a divalent cation [3]. Mn^2+^ was found to be the ion that most effectively enhances the enzyme’s activity, although Mg^2+^ can act as a significant substitute by providing up to 58–75% of the enzyme’s maximal activity. Whereas the *Mf*IDH activity is completely inhibited by Ca^2+^ and Cu^2+^, it is entirely restored by the addition of 2 mM of Mn^2+^ (data not shown). Although most IDHs are strongly inhibited by Zn^2+^, we observed a very interesting effect that Zn^2+^ addition has on *Mf*IDH activity. NAD^+^-linked activity at pH 8.5 was strongly inhibited by Zn^2+^ but partially restored at pH 6.0.

**Table 2:** Effect of metal ions on the activity of *Mf*IDH.

Metal ions	Relative activity (%)	
	NAD^+^	NADP^+^
None	18.0 ± 3.0	17.0 ± 3.5
Mn^2+^	100.0 ± 2.9*	100.0 ± 3.7*
Mg^2+^	75.0 ± 1.5	58.0 ± 2.0
Ca^2+^	0	0
Cu^2+^ (pH 8.5)	10.0 ± 3.5	8.0 ± 2.5
Cu^2+^ (pH 6.0)	0	0
Zn^2+^ (pH 8.5)	3.5 ± 2.5	100.0 ± 3.0
Zn^2+^ (pH 7.0)	25.0 ± 2.0	25.0 ± 2.5
Zn^2+^ (pH 6.0)	50.0 ± 1.5	50.0 ± 2.7

Activity of pure *Mf*IDH was determined with 2 mM metal ions in the standard reaction mixture at pH optimum, unless otherwise specified.

* A 100% activity corresponds to 182 U mg ^-1^ with NAD^+^ and 63 U mg ^-1^ with NADP^+^.

In contrast, the NADP^+^-linked activity at pH 8.5 was fully activated by the presence of Zn^2+^ but decreased by half at pH 6.0, similar to the NAD^+^-linked activity. Thus, the pH optimum of the NADP^+^-linked *Mf*IDH activity drastically changed from pH 6.0 (Mn^2+^) to pH 8.5 (Zn^2+^). Interaction with Zn^2+^ can modulate the *Mf*IDH activity in an interesting manner. Because all the metal binding sites are highly conserved in *Mf*IDH (Fig 2), there is no plausible explanation for these phenomena.

### Effects of analogous cofactors on the *Mf*IDH activity

The effects of different cofactor analogs on the *Mf*IDH activity were examined (Table 3). More than half of the NAD^+^- or NADP^+^-linked activity was retained when the amide group of the nicotinamide ring was replaced by the acetyl group in 3-acetylpyridine adenine dinucleotide (phosphate); thus, the amide group is not indispensable for binding. In contrast, the substitution of the N^6^ amino group of the adenine ring with the oxo-group in nicotinamide hypoxanthine dinucleotide (phosphate) completely abolished NAD(P)^+^ binding. To our knowledge, this is the first report that demonstrates the indispensability of the amino group of adenine in cofactor recognition. Imada et al. thoroughly studied amino acid residues that are involved in the recognition of the adenine and nicotinamide rings of the cofactor [43]. The adenine N^6^ atom is hydrogen-bonded with the carbonyl oxygen of Asn-348 and has amino–aromatic hydrogen-bond interactions with the imidazole ring of His-335, which are conserved interactions in the type I IDHs (Fig 2).

**Table 3:** Effect of cofactor analogous on the activity of *Mf*IDH.

Cofactor	Relative activity (%)
NAD^+^	100.0 ± 2.5*
APAD^+^	55.0 **±** 2.5
NHD^+^	4.0 **±** 1.2
NADP^+^	100.0 **±** 2.8*
APADP^+^	60.0 **±** 2.5
NHDP^+^	2.0 **±** 1.9

APAD(P)^+^, 3-Acetylpyridine adenine dinucleotide (Phosphate); NHD(P)^+^, Nicotinamide hypoxanthine dinucleotide (Phosphate). Activity of pure *Mf*IDH was determined with 0.4 mM cofactor analogous in the standard reaction mixture at pH optimum.

* A 100% activity corresponds to 182 U mg ^-1^ with NAD^+^ and 63 U mg ^-1^ with NADP^+^.

### Substrate specificity and inhibition

No appreciable effect on the activity of *Mf*IDH was observed upon addition of the following compounds (at final concentrations of 5 mM, unless noted) to the reaction mixture: glutamate, glutamine, α-ketoglutarate, oxaloacetate, cis-aconitate, citrate, pyruvate, malate, fumarate, succinate, ADP (2 mM), AMP (2 mM), CoA, AcCoA, NADH, and NADPH (at a final concentration of 0.2 mM). ATP (2 mM) caused 50% inhibition of only the NADP^+^-linked *Mf*IDH activity. Thus, *Mf*IDH activity is not regulated at the metabolic level, as has been demonstrated for IDHs from organisms with a complete TCA cycle.

## Conclusions

The isocitrate dehydrogenase from *M*. *flagellatus* was purified, overexpressed and characterized in the present study. Our data reveal that *Mf*IDH exhibits unique double coenzyme specificity toward both NAD^+^ and NADP^+^ cofactors, and its activity is dependent on divalent cations. *Mf*IDH exhibits a strict cofactor-dependent pH-activity profile. Our study also shows that *Mf*IDH is remarkably thermostable and is not regulated at the metabolic level. We suggest the major amino acids in the protein structure of *Mf*IDH that determine the double cofactor specificity. The enzymatic characterization of *Mf*IDH can enrich our knowledge of type I IDHs and might be useful for the engineering of IDHs with desirable specificities.

## Supporting information

S1 Fig Isoelectric focusing of the native *Mf*IDH. The determination of the isoelectric point of the native *Mf*IDH. M, pI markers; lane 1, purified protein.
(TIFF)

S1 Table Summary of the purification of the native *Mf*IDH. (DOCX)
