# Supplementary figures and images for: Biochemical and molecular characterization of the isocitrate dehydrogenase with dual coenzyme specificity from the obligate methylotroph Methylobacillus Flagellatus
Source: PLoS One. 2017 Apr 19;12(4):e0176056. doi: 10.1371/journal.pone.0176056 (PMC5397045; doi:10.1371/journal.pone.0176056)

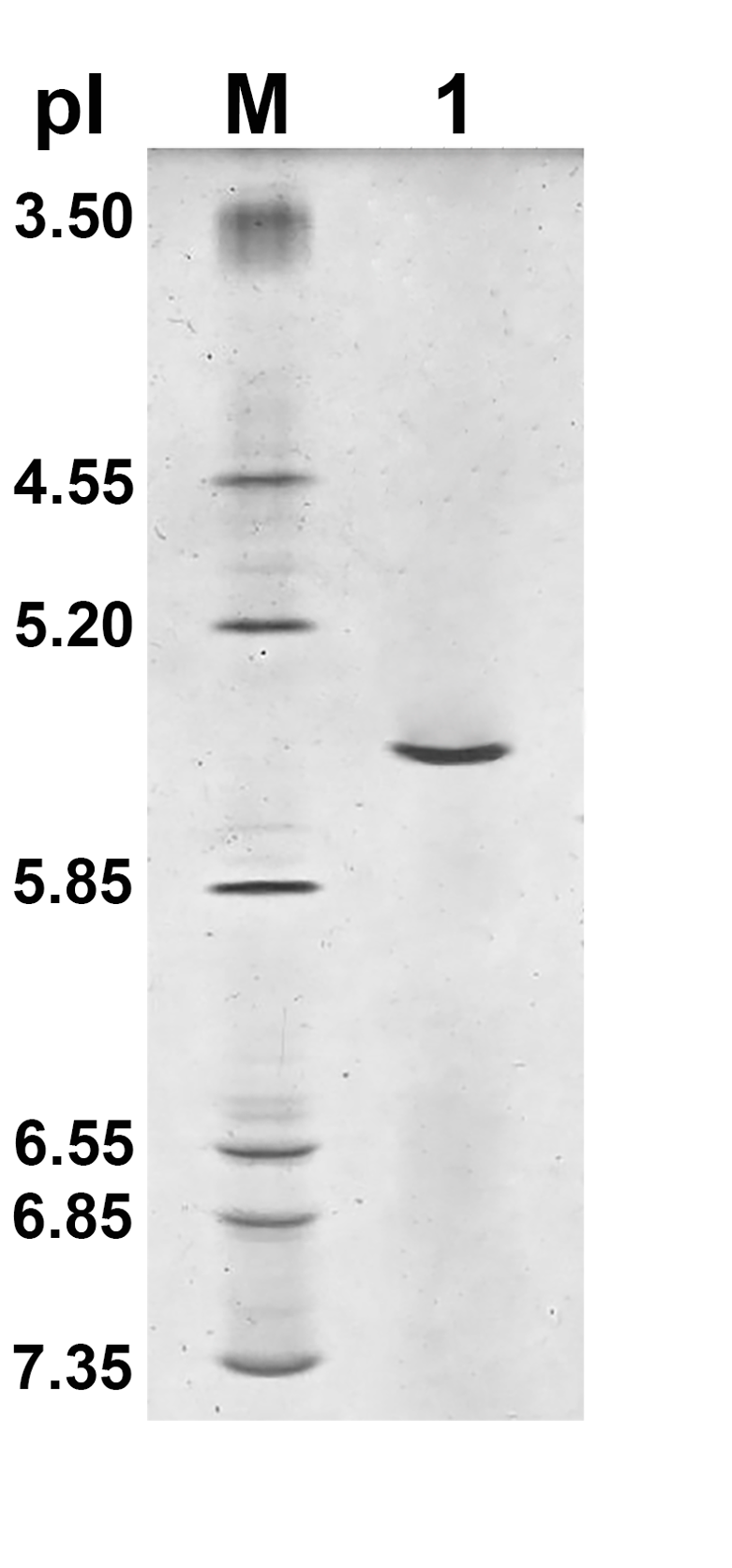

Supplement: S1 Fig — The determination of the isoelectric point of the native MfIDH. M, pI markers; lane 1, purified protein. (TIFF) [file pone.0176056.s001.tiff]
