# Supplementary material for: Biochemical and molecular characterization of the isocitrate dehydrogenase with dual coenzyme specificity from the obligate methylotroph Methylobacillus Flagellatus
Source: PLoS One. 2017 Apr 19;12(4):e0176056. doi: 10.1371/journal.pone.0176056 (PMC5397045; doi:10.1371/journal.pone.0176056)
Supplement: S1 Table — (DOCX) [file pone.0176056.s002.docx]

| **Step** | **Total protein** | **Total activity (U)** | | **Specific activity (U mg ^-1^)** | | **Purification** | **Recovery** |
| --- | --- | --- | --- | --- | --- | --- | --- |
|  | **(mg)** | **NAD^+^** | **NADP^+^** | **NAD^+^** | **NADP^+^** | **(fold)** | **(%)** |
| Crude extract | 1826 | 1425 | 493 | 0.78 | 0.28 | 1 | 100 |
| Heat treatment | 1015 | 1320 | 457 | 1.3 | 0.45 | 1.7 | 93 |
| (50-80 %) (NH_4_)_2_SO_4_ fractionation | 460 | 1150 | 399 | 2.5 | 0.87 | 3.2 | 81 |
| Sephacryl S-100 Gel filtration | 110 | 803 | 278 | 7.3 | 2.5 | 7.3 | 56 |
| Red-Sepharose CL-6B chromatography | 8 | 728 | 252 | 91 | 32 | 116 | 51 |
| PBE-94 chromatofocusing | 3.6 | 655 | 227 | 182 | 63 | 232 | 46 |
